# The Dwarf Phenotype in GH240B Mice, Haploinsufficient for the Autism Candidate Gene *Neurobeachin*, Is Caused by Ectopic Expression of Recombinant Human Growth Hormone

**DOI:** 10.1371/journal.pone.0109598

**Published:** 2014-10-15

**Authors:** Kim Nuytens, Krizia Tuand, Quili Fu, Pieter Stijnen, Vincent Pruniau, Sandra Meulemans, Hugo Vankelecom, John W. M. Creemers

**Affiliations:** 1 Department of Human Genetics, KU Leuven, Leuven, Belgium; 2 Leuven Autism Research Consortium (LAuRes), KU Leuven, Leuven, Belgium; 3 Department of Development and Regeneration, KU Leuven, Leuven, Belgium; Hosptial Infantil Universitario Niño Jesús, CIBEROBN, Spain

## Abstract

Two knockout mouse models for the autism candidate gene *Neurobeachin (Nbea)* have been generated independently. Although both models have similar phenotypes, one striking difference is the dwarf phenotype observed in the heterozygous configuration of the GH240B model that is generated by the serendipitous insertion of a promoterless *human growth hormone* (*hGH*) genomic fragment in the *Nbea* gene. In order to elucidate this discrepancy, the dwarfism present in this *Nbea* mouse model was investigated in detail. The growth deficiency in *Nbea* ^+/−^ mice coincided with an increased percentage of fat mass and a decrease in bone mineral density. Low but detectable levels of hGH were detected in the pituitary and hypothalamus of *Nbea* ^+/−^ mice but not in liver, hippocampus nor in serum. As a consequence, several members of the mouse growth hormone (mGH) signaling cascade showed altered mRNA levels, including a reduction in growth hormone-releasing hormone mRNA in the hypothalamus. Moreover, somatotrope cells were less numerous in the pituitary of *Nbea* ^+/−^ mice and both contained and secreted significantly less mGH resulting in reduced levels of circulating insulin-like growth factor 1. These findings demonstrate that the random integration of the *hGH* transgene in this mouse model has not only inactivated *Nbea* but has also resulted in the tissue-specific expression of hGH causing a negative feedback loop, mGH hyposecretion and dwarfism.

## Introduction

*NEUROBEACHIN* (*NBEA*) has previously been identified as a candidate gene for autism spectrum disorder (ASD) based on associations and linkage studies and patients with monoallelic inactivation of NBEA [1]–[8].

To further investigate the function of NBEA, *Nbea* heterozygous and knockout mouse models have been used. Independently, two models have been generated in which the *Nbea* gene is disrupted [9], [10]. The first model was serendipitously generated via random integration of a construct containing a minimal human *growth hormone* (h*GH*) genomic fragment. In the GH240B transgenic line this insertion occurred in intron 1 of *Nbea* leading to a genuine null allele [10]. Expression of the h*GH* minigene was examined and could not be detected. The second *Nbea* mouse model was generated by means of the gene-trap vector system (further referred to as the gene-trap mouse) [9]. Both mouse models show similar phenotypes in which bi-allelic inactivation of Nbea results in neonatal paralysis and death. This is caused by a complete block of evoked neuromuscular synaptic transmission as was shown in the GH240B *Nbea^−/−^* mice [10]. The GH240B *Nbea^+/−^* mice also revealed abnormal morphology of dense-core granules in blood platelets, similar to the first described patient with NBEA haploinsufficiency. Furthermore, a decrease in several actin-associated peptides was observed. In addition, these studies in blood platelets confirmed the important role of NBEA as an A-kinase anchoring protein, affecting phosphorylation levels of several PKA substrates [11]. Increased BDNF levels in the hippocampus of these mice are probably caused by an increased CREB phosphorylation level as described in β-TC3 cells. Behavioral studies in this mouse model showed an autism-like phenotype with increased repetitive self-grooming behavior, increased conditioned fear, decreased sociability and a delayed spatial learning and memory [12]. Studies in the gene-trap mouse models showed that loss of NBEA leads to an imbalance in excitatory and inhibitory signaling with a more affected inhibitory network, an ectopic accumulation of F-actin in the neuronal cell body, the dendritic shafts and axons and a decrease in surface levels of GABA_A_ and glutamate receptors [9], [13], [14]. Reduced glutamate responsiveness was measured and confirmed in both lines [13].

One striking difference between both mouse models is the presence of a dwarf phenotype in *Nbea* ^+/*−*^ mice. This growth deficiency is only present in the GH240B *Nbea* ^+/*−*^ mouse model [10] whereas the gene-trap *Nbea* ^+/*−*^ mice have a normal stature accompanied by a higher body weight due to an increased adipose tissue mass [15].

In order to elucidate this discrepancy, the dwarf phenotype of GH240B *Nbea* ^+/*−*^ mice was investigated in detail. A growth curve was generated and the expression of genes in the GH pathway was measured at mRNA and protein level. Alterations in body composition were detected via dual-energy X-ray absorptiometry (DEXA). Pituitary cell numbers were determined and pituitary size and the ultrastructure of somatotrope cells were examined. The responsiveness of somatotrope cells to secretagogues was analyzed in *ex vivo* cultures of anterior pituitaries. Finally, hGH expression, which was previously found to be undetectable, was re-examined in serum, pituitary, hypothalamus, hippocampus and liver.

## Materials and Methods

### Animals

The GH240B transgenic line [10] was backcrossed for at least 10 generations with C57BL/6JRj mice (Janvier). Mice were housed in standard cages (wood-shaving bedding) under conventional laboratory conditions (12/12h light/dark cycle, lights on at 8am; 22°C) and were allowed food and water *ad libitum*. All experiments were performed with 12 week old male mice unless otherwise stated.

### Ethics statement

All experiments were approved by the ethical research committee of KU Leuven in accordance with the declaration of Helsinki (project number P182/2011).

### Assessment of a growth curve

Mice were weighed weekly starting at postnatal day 2 until mice reached adulthood (12 weeks of age). Afterwards, mice were weighed once a month until they were 1 year of age. In total 10 male *Nbea* ^+/+^ mice and 17 male *Nbea* ^+/*−*^ mice were included.

### Whole body dual-energy X-ray absorptiometry (DEXA)

Mice were anesthetized by intraperitoneal injection with 10 mg/kg Xylazine (Rompun, Bayer, Germany) and 100 mg/kg Ketamine (Ketamine 1000 CEVA, Ceva Sante Animal, Belgium). The body composition of anesthetized mice (n = 5/genotype) was measured by DEXA (PIXImus densitometer, Lunar Corp., Madison, USA) using ultra-high resolution (0.18×0.18 pixels, resolution of 1.6 line pairs/mm) and software version 1.45.

### RNA extraction and quantitative reverse transcription PCR (qRT-PCR)

RNA of the anterior pituitary, hypothalamus and liver was extracted with the Nucleospin RNA XS or L kit (Macherey-Nagel, Germany) according to the manufacturer's protocol. Per experiment, an equal amount of RNA was reverse transcribed to cDNA using the iScript cDNA synthesis kit (Bio-Rad, California, USA). Real-time qRT-PCR was performed using iQ SYBR Green Supermix (Bio-Rad). 40 cycles of annealing/extension for 1 min at 60°C were carried out with the MyiQ single color real-time PCR detection system (Bio-Rad). The relative amount of mRNA expression was calculated using the method of Livak with normalization to glyceraldehyde 3-phosphate dehydrogenase (GAPDH) as reference gene [16]. The primers used are given as supporting information (Table S1).

### Quantification of growth hormone (GH) and insulin-like growth factor 1 (IGF-1)

Murine (m)GH levels were quantified using the Rat/Mouse Growth hormone ELISA (Millipore, Darmstadt, Germany) according to the supplier's protocol. The content of human (h)GH was calculated by means of the HGH human direct ELISA kit (Invitrogen, Paisley, UK) according to the manufacturer's instructions. The plasma samples measured were derived from blood taken at 3 different time points to cover the peak of GH release in the mouse, namely 09:30 h, 10:00 h and 10:30 h [17]. Hippocampi were dissected and immediately put on −80°C. Tissue was homogenized in sucrose buffer (3.18 mM sucrose; 4 mM Tris-HCl pH 7.4) containing a protease and phosphatase inhibitor cocktail (Roche Diagnostics GmBH, Mannheim, Germany). IGF-1 levels in plasma, derived from the blood samples taken at 10:30 h, (n = 3/genotype) were measured using the Quantakine ELISA Mouse/Rat IGF-1 Immunoassay (R&D systems, Minneapolis, USA) according to the supplier's guidelines.

### Transmission electron microscopy (TEM) of somatotrope cells

Mice were anesthetized with an intraperitoneal injection of 50 mg/kg Nembutal (CEVA Sante Animale, Brussels, Belgium). After gravitational perfusion of the mice with Karnovsky's fixative (2.5% glutaraldehyde, 2% formaldehyde in 0.1 M sodium cacodylate buffer; pH 7.4), pituitary glands were isolated and additionally fixed for 2 hours at room temperature, followed by an overnight immersion at 4°C in Karnovsky's fixative. After the samples were washed and postfixed with 1% OsO4, they were dehydrated through a series of increasing ethanol concentrations and embedded in epoxy resin. Ultrathin sections (70 nm) were collected on formvar-coated copper grids and stained with lead citrate and uranyl acetate.

TEM of adenopituitary tissue sections was performed at 80 kV using a JEM1400 transmission electron microscope (JEOL, Tokyo, Japan). All micrographs were acquired on a SIS Quemesa camera with the same spatial resolution. One pixel corresponds to 12.44 nm. Somatotropes were identified based on their morphological description as defined in [18].

### Quantification of the different hormonal cell types in the anterior pituitary

This experiment was performed as described before [19]. Briefly, naive mice were euthanized by CO_2_ asphyxiation followed by immediate decapitation. The neural and intermediate lobes of the pituitary were discarded using a stereomicroscope. The anterior lobe (anterior pituitary) was isolated and dissociated into individual cells with trypsin. Cells were resuspended in serum-free defined medium (SFDM; Invitrogen) and spun down on Cytospin slides (SuperfrostPlus; Thermo Scientific, Rockford, USA). Cells were permeabilized with 0.5% saponin (Sigma-Aldrich, St. Louis, USA) after fixation in 4% paraformaldehyde, and were incubated overnight with antibodies against murine GH (1/10,000), prolactin (PRL) (1/10,000), adrenocorticotropic hormone (ACTH) (1/5,000) and α-glycoprotein subunit (αGSU) (1/1,000). Hormone antisera were obtained from Dr. A. F. Parlow (National Hormone and Peptide Program, Harbor-UCLA Medical Center, Torrance, CA). Subsequently, cells were incubated for 1 h with fluorescently labeled secondary antibodies (Invitrogen; 1/1,000) and 6-diamidino-2-phenylindole (DAPI). Images were captured using an Olympus IX81 microscope. Cells were counted using ImageJ (NIH, Maryland, USA) in at least 5 random cytospin fields (1,000–2,000 cells per hormone per group) and the proportions of immunopositive cells on the total anterior pituitary cells were determined. To take into account the smaller size of the anterior pituitary and the correspondingly lower number of total anterior pituitary cells (about 2-fold, see Results) in the *Nbea^+/−^* GH240B mice, we calculated the decrease in absolute cell number of the different cell types (using their proportion and the total number of anterior pituitary cells obtained per group of mice).

### Primary anterior pituitary cell culture and regulated secretion assay

Primary cultures were generated based on the protocol described [19]. Briefly, anterior pituitaries were dissected and dissociated into single cells as reported above. Cells were resuspended in SFDM, quantified using a Z2 Coulter counter (Analis, Namur, Belgium), and seeded in cell culture plates with Primaria surface treatment (BD Biosciences, Bergen County, USA). Basal GH secretion was defined as the content GH in SFDM without secretagogues after 2 h incubation, normalized for the total amount of GH in medium samples plus lysates. cAMP-induced GH release was measured after a 2 h incubation in SFDM containing the non-specific inhibitor of phosphodiesterases 3-isobutyl-1-methylxanthine (IBMX; 100 µM) and the adenylate cyclase activator Forskolin (1 µM). Stimulated secretion was defined as the amount of GH secreted after stimulation with IBMX/Forskolin minus basal release and normalized for the total amount of GH in medium samples plus lysates. Afterwards, cells were lysed in RIPA buffer (25 mM Tris-HCl pH 7.6; 150 mM NaCl; 1% NP-40; 1% sodium deoxycholate; 0.1% sodium dodecyl sulfate) containing a protease and phosphatase inhibitor cocktail.

### Immunoblotting

Anterior pituitary, hypothalamus and liver were homogenized in RIPA buffer containing a protease and phosphatase inhibitor cocktail. Anterior pituitaries and hypothalami of littermates were pooled and homogenization was performed manually using a pellet pestle for 1.5 ml tubes. Liver tissue was homogenized with a motor-driven Potter-Elvehjem tissue grinder (Wheaton, Millville, USA). The protein concentration of the samples was quantified with a BCA protein assay (Thermo Scientific, Rockford, USA). 25 µg of lysate was loaded on a 10% Bis-Tris gel (Bio-Rad, California, USA). Proteins were transferred to Protran Nitrocellulose membrane (Schleicher&Schuell, Dassel, Germany) and incubated with antibodies against human GH (hGH) (Abcam, Cambridge, UK; 1/1,000) and actin (Sigma-Aldrich; 1/5,000), used for normalization. Afterwards, membranes were incubated with HRP-conjugated secondary antibody (1/2,000; Dako, Glostrup, Denmark) and proteins were visualized with western blotting ECL detection reagent.

### Immunofluorescent staining of human GH in total pituitary

Total pituitaries were dissected, followed by an overnight fixation in 4% formaldehyde. After rinsing in 70% ethanol, pituitaries were embedded in paraffin according to standard procedures. Tissue blocks were cut in 5 µm sections with subsequent removal of paraffin with Histo-Clear (National Diagnostics, Hessle, UK) and dehydration through serial ethanol solutions. Preceding a 30 min block in 10% goat serum in PBS with 2% BSA, sections were incubated for 20 min in target retrieval solution, pH 6 (Dako). Sections were incubated for 1.5 h with mouse anti-hGH antibody (Abcam; 1/1,000) and subsequently for 1 h with Alexa 488–labeled anti-mouse antibody (Molecular probes, Paisley, UK; 1/500) before mounting in ProLong Gold Antifade Reagent (Dako) containing DAPI. Immunofluorescence was visualized by Axiovision software (Zeiss, Heidelberg, Germany) with the Axio Imager Z1 microscope (Zeiss).

### Statistical analysis

Data are presented as mean with standard error of the mean (SEM). Significance of differences between mean values were analyzed using (where appropriate) two-tailed *t* test, analysis of variance (ANOVA) or repeated measures (RM)-ANOVA procedures with Tukey test *post hoc* comparison using Statistica version 9.0 (StatSoft, Oklahoma, USA). All statistical tests were performed with 0.05 as the α level of significance (*: *P*<0.05, **: *P*<0.01 and ***: *P*<0.001).

## Results

### Reduced body weight of mice heterozygous for *Nbea*

**Figure 1:**
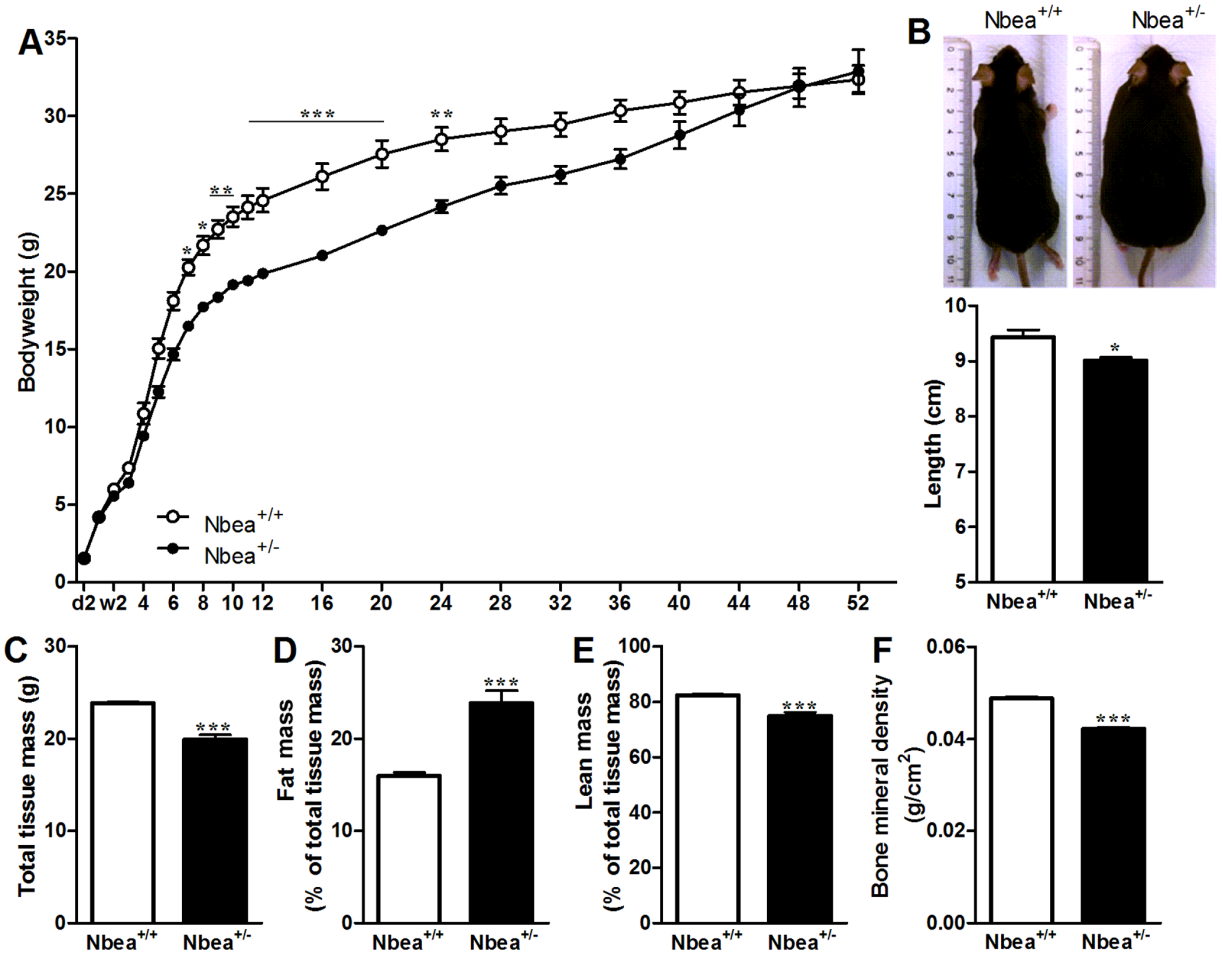
*Nbea* ^+/^^*−*^ mice show postnatal, sustained growth retardation and an altered body composition. (**A**) Male mice were weighed from postnatal day 2 until week 52. (**B**) Male mice were sacrificed at 1 year of age revealing a reduced body length of *Nbea* ^+/*−*^ mice compared to *Nbea* ^+/+^ mice (*Nbea* ^+/+^ mice: n = 10, *Nbea* ^+/*−*^ mice: n = 17). (**C–F**) DEXA measurements were performed to analyze the body composition (n = 5/genotype; 12 weeks old). (**C**) *Nbea* ^+/*−*^ mice have a reduced total tissue mass accompanied by an increase in percentage fat mass (**D**) and a decreased percentage lean mass (**E**). (**F**) Also, the bone mineral density of *Nbea* ^+/*−*^ mice was significantly reduced in comparison with *Nbea* ^+/+^ mice. *: *P*<0.05, **: *P*<0.01; ***: *P*<0.001.

To characterize the growth retardation of *Nbea* ^+/*−*^ GH240B mice, their body weight was measured from postnatal day 2 until mice were 1 year old (Figure 1A). The body weight of *Nbea* ^+/*−*^ mice was initially significantly lower than the weight of their wild-type littermates (RM-ANOVA: main effect of genotype: F(1,25) = 17.3, *P*<0.001). This dwarf phenotype was apparent after 4 weeks and became statistically significant starting 7 weeks after birth and persisted into adulthood. As mice became older, *Nbea* ^+/*−*^ mice showed an increased weight gain in comparison to *Nbea* ^+/+^ mice (RM-ANOVA: interaction genotype x age: F(22,550) = 8.4, *P*<0.001) (Figure 1A). At 1 year of age, mice were sacrificed and their body length was measured. Heterozygous mice were significantly smaller than their wild-type littermates (*t* = 3.39, *P* = 0.002), despite similar weight (Figure 1B).

### Different body composition of *Nbea* ^+/+^ and *Nbea* ^+/*−*^ mice

A significant interaction effect of genotype with age in the body weight curve might be indicative for alterations in fat percentage of *Nbea* ^+/*−*^ mice. Therefore, the body composition of adult mice was determined by DEXA. Although the total tissue mass was significantly lower in *Nbea^+/−^* mice than in *Nbea* ^+/+^ mice (*t* = −7.91, *P*<0.001) (Figure 1C), *Nbea* ^+/*−*^ mice contain an increased fat mass percentage and an accordingly decreased lean mass percentage (%fat: *t* = 5.50, *P*<0.001; %lean: *t* = −5.3, *P*<0.001) (Figure 1D and E). In addition, the bone mineral density of *Nbea* ^+/*−*^ mice was significantly lower compared to *Nbea* ^+/+^ mice (*t* = −13.1, *P*<0.001) (Figure 1F).

### The GH/IGF-1 axis is affected in *Nbea* ^+/*−*^ mice

**Figure 2:**
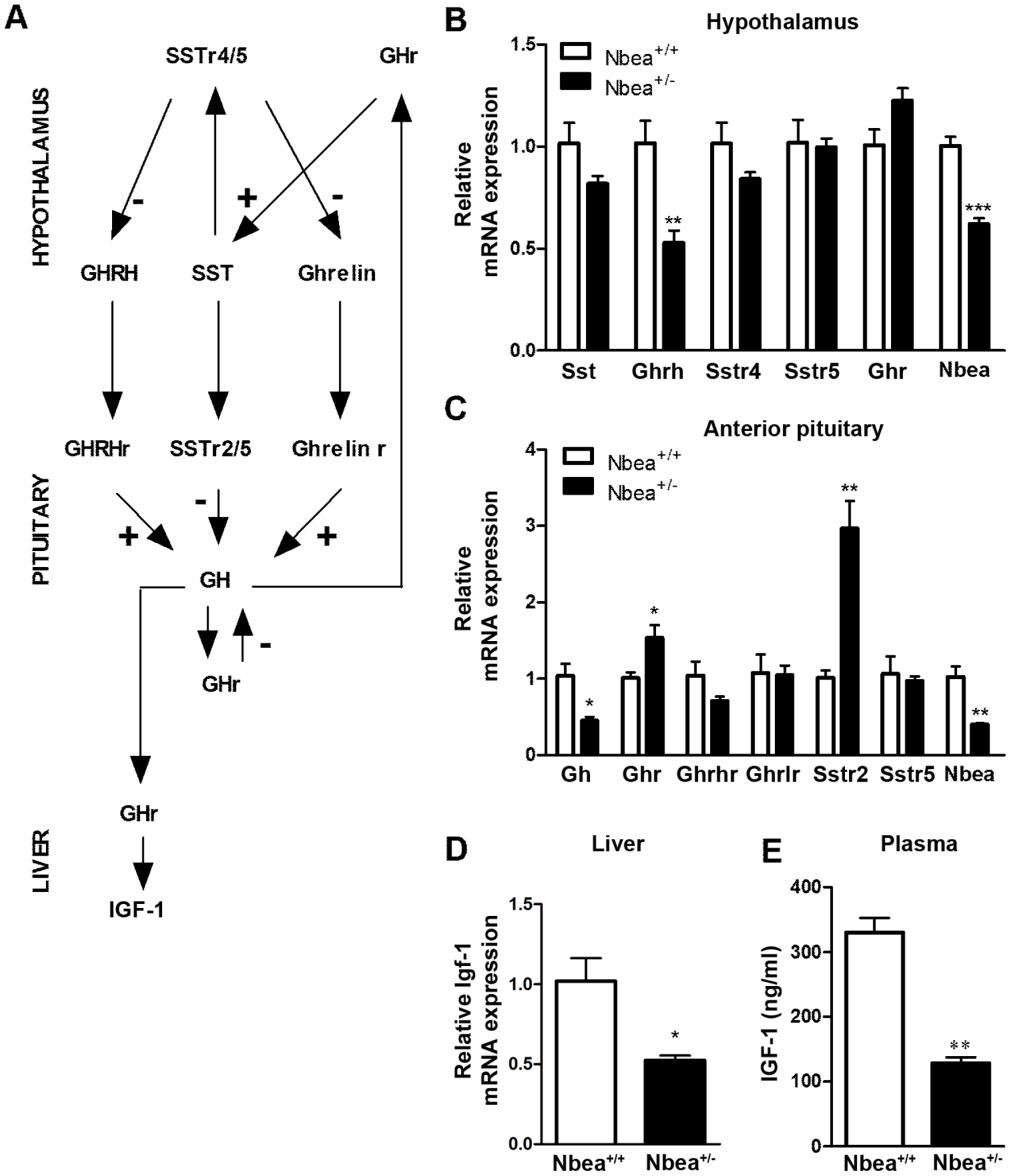
Alterations in the GH signaling cascade of *Nbea* ^+/^^*−*^ mice. (**A**) Schematic representation including the members of the GH pathway of which the mRNA level was measured by qRT-PCR. Black arrows indicate receptor binding, a + or – added to the arrow implicates a stimulation or inhibition effect, respectively. (**B**) In the hypothalamus of *Nbea* ^+/*−*^ mice, the expression level of *Ghrh* was significantly reduced. The mRNA level of *Ghrelin* was too low to generate conclusive data (n = 4 litters/genotype; 12 weeks old). (**C**) In the anterior pituitary, the mRNA level of *Gh* was significantly decreased in *Nbea* ^+/*−*^ mice. Moreover, the expression level of *Ghr* and *Sstr2* was significantly increased in *Nbea* ^+/*−*^ mice (n = 4 litters/genotype; 12 weeks old). (**D**) The mRNA level of *Igf-1* was significantly reduced in the liver of *Nbea* ^+/*−*^ mice (n  =  3/genotype; 12 weeks old). (**E**) The reduced mRNA level is reflected in a decrease of plasma IGF-1 in *Nbea* ^+/*−*^ mice (n = 3/genotype; 12 weeks old). *Gh: growth hormone; Ghr: Gh receptor; Ghrh: Gh-releasing hormone; Ghrhr: Ghrh receptor; Sst: somatostatin; Sstr: Sst receptor; Ghrlr: Ghrelin receptor; Igf-1: insulin-like growth factor 1;* *: *P*<0.05, **: *P*<0.01; ***: *P*<0.001.

The observed dwarf phenotype of *Nbea* ^+/*−*^ mice in combination with the altered body composition suggested abnormalities in the GH signaling cascade. To assess alterations in the GH/IGF-1 axis, mRNA expression of the relevant genes was analyzed in anterior pituitaries, hypothalami and livers from adult mice (Figure 2A). In the hypothalamus, a significant reduction in *Gh-releasing hormone* (*Ghrh*) expression was detected (*t* = 3.91, *P* = 0.008) (Figure 2B). The expression level of *Ghrelin* was too low to be conclusive. The expression levels of *somatostatin* (*Sst*), *Sst receptors* (*Sstr*) *4* and *5*, and *Gh receptor* (*Ghr*) did not significantly differ between genotypes (*Sst*: *t* = 1.85, *P* = 0.11; *Sstr4*: *t* = 1.62, *P* = 0.15; *Sstr5*: *t* = 0.18, *P* = 0.86; *Ghr*: *t* = −2.29, *P* = 0.07). In the anterior pituitary, a significant decrease in *Gh* mRNA expression was accompanied by a significant increase in *Ghr* and the most abundantly expressed somatostatin receptor *Sstr2* mRNA expression (*Gh*: *t* = 3.55, *P* = 0.012; *Ghr*: *t* = −2.96, *P* = 0.025, *Sstr2*: *t* = −5.19, *P* = 0.002) (Figure 2C). No significant differences in the expression level of *Ghrh receptor* (*Ghrhr*), *Ghrelin receptor (Ghrlr)* and *Sstr5* were detected (*Ghrhr*: *t* = 1.71, *P* = 0.14; *Ghrlr*: *t* = 0.09, *P* = 0.86; *Sstr5*: *t* = 0.41, *P* = 0.70). One of the main consequences of peripheral GH signaling is the production of IGF-1 in the liver [20]. Therefore, *Igf-1* mRNA levels in the liver were determined, combined with the quantification of IGF-1 present in plasma. A significant decrease in *Igf-1* mRNA in the liver was detected (*t* = 3.37, *P* = 0.028) which resulted in a significantly reduced level of IGF-1 in the plasma (*t* = 8.36, *P* = 0.001) (Figure 2 D and E).

### Reduced presence and GH content of somatotrope cells in *Nbea^+/−^* mice

**Figure 3:**
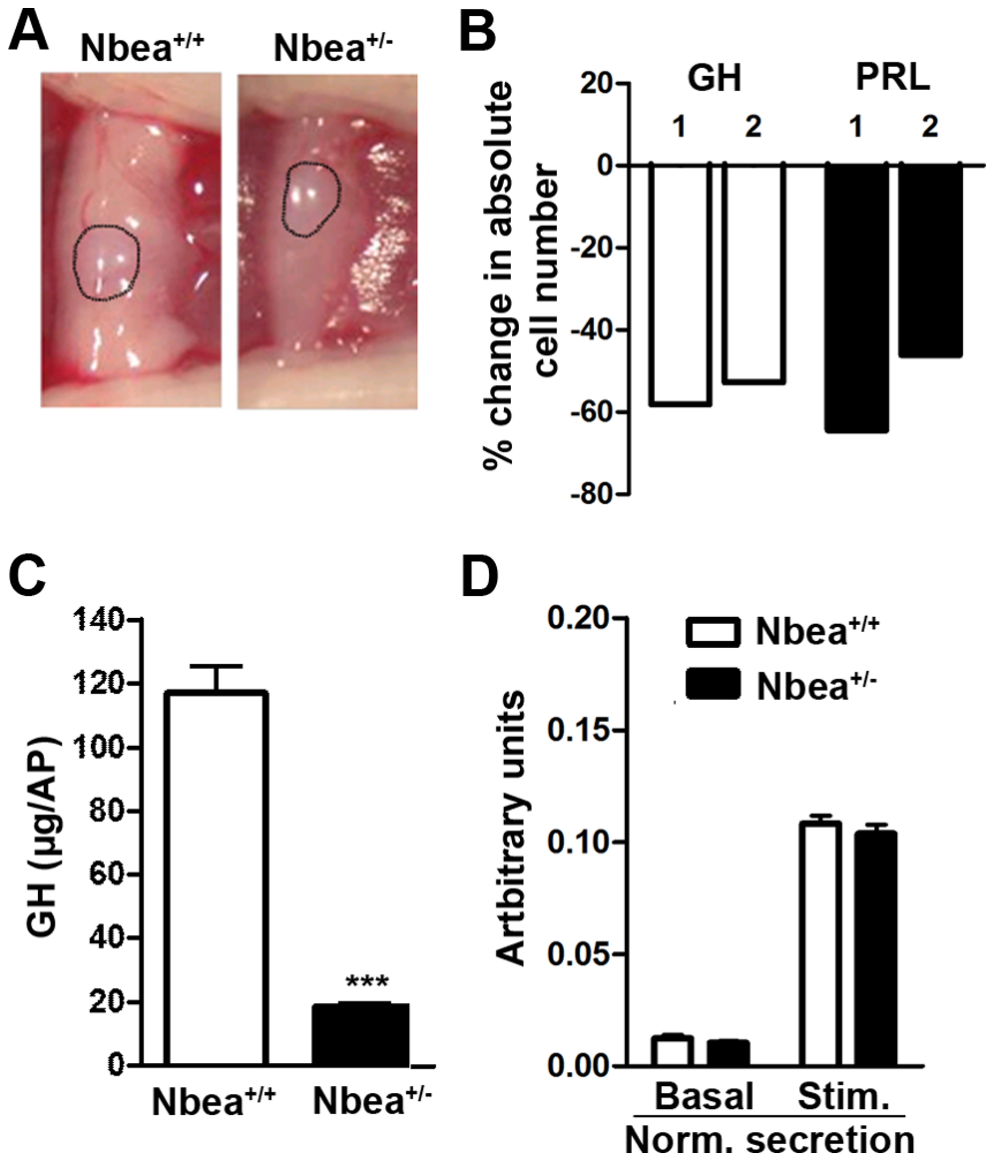
Somatotrope cells in *Nbea* ^+/^^*−*^ mice are less numerous and contain less GH than **Nbea** ^+/+^ mice. (**A**) The pituitary of *Nbea^+/−^* mice is smaller. More specifically, the anterior pituitary is affected since the size of the posterior pituitary (dashed line) appears to be unaffected. (**B**) Percent change in absolute number of growth hormone (GH) and prolactin (PRL) immunopositive cells in the anterior pituitary of *Nbea^+/−^* compared to control mice, shown for 2 experiments separately (1,000–2,000 cells per hormone were counted per genotype; the experiment was conducted twice on 12 weeks old male mice). (**C**) In addition to the reduced presence of somatotrope cells in the anterior pituitary of *Nbea* ^+/*−*^ mice, they also contained significantly less GH (n = 3/genotype; 12 weeks old). (**D**) Primary anterior pituitary cell cultures of *Nbea^+/−^* mice secrete similar to *Nbea^+/+^* mice under basal conditions and upon stimulation with IBMX and Forskolin (Stim.), after we normalized the GH content in the medium for the total GH level in somatotropes (n = 4/genotype). *: *P*<0.05, **: *P*<0.01; ***: *P*<0.001.

During microdissection of the anterior pituitaries, abnormal morphology of the pituitary of *Nbea* ^+/*−*^ mice became apparent (Figure 3A). The size of the anterior pituitary seemed to be reduced in *Nbea^+/−^* mice, while the size of the neuropituitary appeared to be unchanged by visual inspection. To assess which cell types are affected, anterior pituitaries were dispersed into single cells, fixed, and marker proteins for specific cell types were fluorescently labeled and immunopositive cells counted. In accordance with the smaller size, a reduction in total anterior pituitary cell number was detected for *Nbea* ^+/*−*^ mice compared to *Nbea* ^+/+^ mice (330,000 and 300,000 cells versus 650,000 and 660,000 cells; n = 2/genotype). Furthermore the cell composition of these anterior pituitaries was assessed and a 55%±3 and 55%±9 decrease in the absolute number of GH- and prolactin- (PRL) positive cells, respectively, was detected in the anterior pituitary of *Nbea* ^+/*−*^ mice when compared to control mice (Figure 3B; n = 2). The number of cells containing adrenocorticotropic hormone (ACTH) and αGSU, which is the α-glycoprotein subunit of the thyroid-stimulating hormone (TSH), follicle-stimulating hormone (FSH) and luteinizing hormone (LH), is low and was therefore too variable to allow firm conclusions.

In addition, the total GH content of the anterior pituitary of *Nbea* ^+/*−*^ and *Nbea* ^+/+^ mice was measured to assess if the GH content of the remaining somatotrope cells was affected. Based on the reduced presence of somatotrope cells by approximately 50%, a similar decrease in GH content per anterior pituitary is expected if the remaining somatotrope cells contain normal amounts of GH. However, in *Nbea^+/−^* mice compared to *Nbea^+/+^* mice, a decline in GH content per anterior pituitary, with a ratio of 1/6.32±0.17 was observed, instead of the expected 2-fold (*Nbea^+/+^* mice: 117.01±8.5 µg GH per anterior pituitary; *Nbea^+/−^* mice: 18.5±1.16 µg GH per anterior pituitary; *t* = 11.48, *P*<0.001; n = 3/genotype) (Figure 3C).

**Figure 4:**
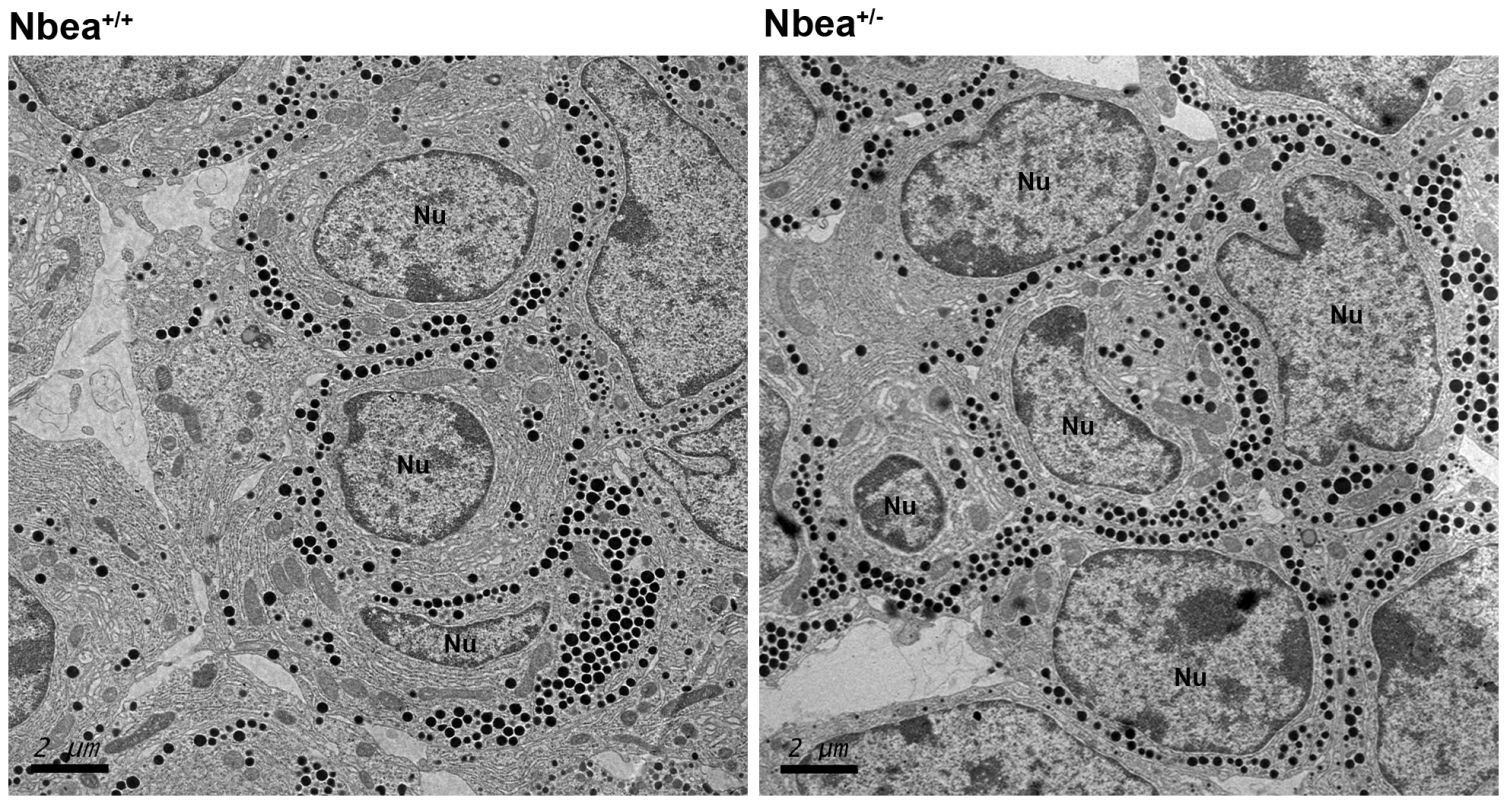
Normal morphology of somatotrope cells of *Nbea* ^+/^^*−*^ mice. Electron microscopic views show representative somatotrope cells of 12 weeks old *Nbea* ^+/+^ and *Nbea* ^+/*−*^ mice with no apparent ultrastructural differences. Nu: nucleus of a somatotrope cell.

To investigate any morphological abnormalities of the GH-producing cells, transmission electron microscopy was performed. However, the ultrastructure of the somatotrope cells and the large electron-dense granules containing GH appeared unaffected (Figure 4).

### Normal responsiveness of the somatotrope cells in *Nbea* ^+/*−*^ mice

In order to evaluate the responsiveness of the remaining somatotrope cells, primary anterior pituitary cell cultures were generated allowing GH measurements under basal conditions and after stimulation with IBMX and Forskolin, secretagogues mimicking the cAMP-stimulating effect of GHRH (Figure 3D) [21]. Primary cultures of *Nbea* ^+/*−*^ mice secreted significantly less GH compared to *Nbea* ^+/+^ mice (ANOVA: main effect of genotype: F(1,18) = 32.3, *P*<0.001 and *post hoc* comparison: basal medium: *P*<0.001 and stimulated medium: *P*<0.001) (Figure 3D). In addition, the remaining GH in the cell lysate was significantly lower in cell cultures derived from *Nbea* ^+/*−*^ mice (ANOVA: interaction genotype x condition: F(2,18) = 21.0, *P*<0.001 and *post hoc* comparison: cell lysate: *P* = 0.003). Consistent with a reduced amount of GH per somatotrope cell in *Nbea* ^+/*−*^ mice, the total GH content of 200,000 anterior pituitary cells, defined as the sum of GH in basal and stimulated medium and the cell lysate, was decreased in *Nbea^+/−^* mice with 71%±3 compared to the total GH content as measured in *Nbea^+/+^* mice (*t* = 5.15, *P* = 0.002), while the reduction of GH cells was only 55% (see above). We analyzed the effect on secretion by normalizing the absolute amount of GH in the medium for their total GH content and found no significant difference for genotype in basal nor in stimulated secretion (two-way ANOVA: genotype: F (1,12) = 1.28, *P* = 0.27; condition: F (1,12) = 1135.9, *P*<0.0001; interaction genotype x condition: F (1,12) = 0.212, *P* = 0.65), indicating a normal responsiveness of GH secretion upon stimulation (Figure 3D). Taken together, *Nbea^+/−^* anterior pituitary cell cultures contain less GH but respond normally to secretagogues.

### Expression of human GH in the pituitary and hypothalamus of *Nbea* ^+/*−*^ mice

The *Nbea* mouse model used in this study was generated serendipitously by integration of a transgenic construct of h*GH* into intron 1 of the *Nbea* gene resulting in a *Nbea* null allele. Although the absence of h*GH* expression in the pituitary was previously shown by RT-PCR [22] a more extensive analysis of hGH expression was conducted here.

**Figure 5:**
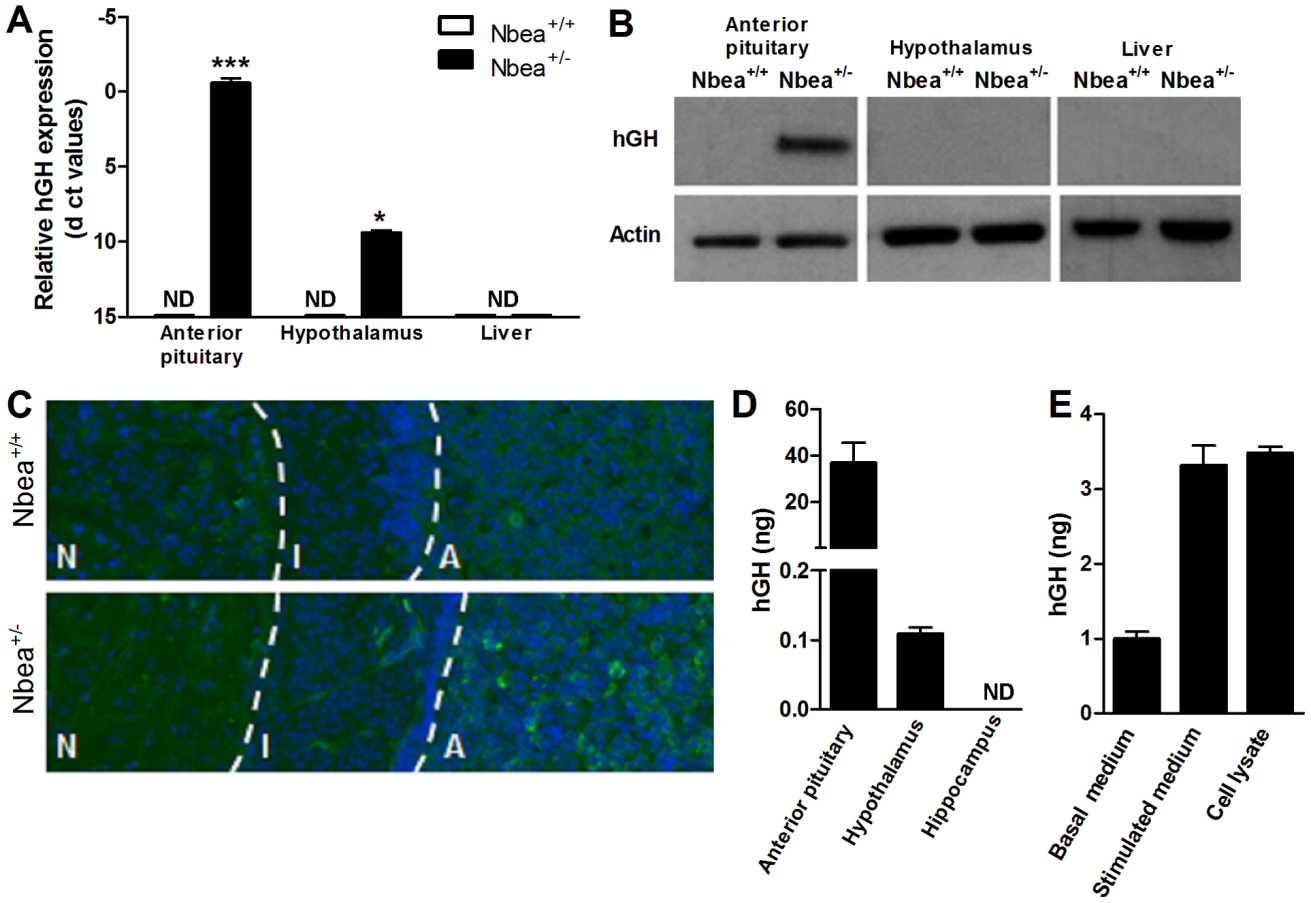
Presence of h*GH* mRNA and protein in the pituitary and hypothalamus of *Nbea* ^+/^^*−*^ mice. (**A**) The qRT-PCR data are represented as Δct values (*Gapdh* expression) [16]. In *Nbea* ^+/*−*^ mice, the highest h*GH* mRNA levels were detected in anterior pituitary followed by the hypothalamus. No signal was detected in liver samples of both *Nbea^+/−^* and control mice (n = 3/genotype). (**B**) Western blot shows that in the anterior pituitary of *Nbea* ^+/*−*^ mice h*GH* protein is present whereas no h*GH* could be detected in the hypothalamus and liver. All tissue from *Nbea* ^+/+^ mice was negative for h*GH* (n = 3/genotype). (**C**) Immunofluorescent staining of hGH gives a positive signal in the anterior (A) and intermediate lobe (I) of the pituitary of *Nbea* ^+/*−*^ mice. No signal was detected in the neural lobe (N) of *Nbea* ^+/*−*^ mice. (**D**) The anterior pituitary of *Nbea* ^+/*−*^ mice contained approximately 40 ng hGH, measured by ELISA for hGH. A significant amount of hGH in the hypothalamus of *Nbea* ^+/*−*^ mice was detected (n = 3/genotype). (**E**) Under basal conditions, a limited amount of hGH was secreted in anterior pituitary cell cultures. Upon stimulation, approximately half of the total amount of hGH was secreted. No signal of hGH could be detected in all wild-type samples (n = 3/genotype). ND: not detectable; *: *P*<0.05, **: *P*<0.01; ***: *P*<0.001.

Relative mRNA expression of h*GH* was determined in different tissues (Figure 5A). In the anterior pituitary of *Nbea* ^+/*−*^ mice, the mRNA level of h*GH* was comparable to *Gapdh* mRNA levels while it was undetectable in *Nbea* ^+/+^ mice (*t* = 13.94, *P*<0.001). Although the h*GH* expression in the hypothalamus was also significantly higher compared to *Nbea* ^+/+^ mice (t = 4.48, *P* = 0.01), the h*GH* expression was much lower in comparison to the anterior pituitary, albeit still clearly detectable. The liver of both *Nbea* ^+/*−*^ and *Nbea* ^+/+^ mice contained at best trace levels of h*GH* mRNA (*t* = 0.14, *P* = 0.89).

To assess whether the mRNA levels correlated with protein levels, a western blot of hGH was performed on lysates of the same tissues (Figure 5B). hGH protein was detected in the anterior pituitary of *Nbea* ^+/*−*^ mice, but not in the hypothalamus and liver. As expected, no hGH could be detected in anterior pituitary, hypothalamus and liver of *Nbea* ^+/+^ mice.

To obtain insight into which lobes of the pituitary express hGH, immunofluorescent staining of hGH was performed on sections of the total pituitary. An immunopositive signal was detected in the anterior and to a lesser extent the intermediate lobe of the pituitary of *Nbea* ^+/*−*^ mice (Figure 5C).

In order to determine the ratio of hGH relative to murine GH (mGH) in the anterior pituitary of *Nbea* ^+/*−*^ mice, an hGH ELISA was performed on anterior pituitary lysates. In addition, lysates of the hypothalamus were included since an ELISA is more sensitive than a western blot. In the anterior pituitary of *Nbea* ^+/*−*^ mice a total of 37±9 ng hGH was present (Figure 5D), leading to an mGH/hGH ratio of 505. In the hypothalamus of *Nbea* ^+/*−*^ mice, hGH was also detected with the sensitive ELISA (0.11±0.01 ng), but at much smaller amounts than in the pituitary which is consistent with mRNA data (Figure 5D). It is worth noting that the signal detected in all wild-type samples was under the detection limit of the hGH ELISA.

To find out whether hGH is more generally expressed in the brain, levels were also measured in lysates of the hippocampus since this region has the highest expression of NBEA. The values detected in both wild-type and heterozygous mice were below the detection limit of the ELISA (Figure 5D).

To analyze whether hGH is secreted under basal and stimulated conditions, the hGH levels were measured in the samples of the primary anterior pituitary cell cultures. Under basal conditions, 0.99±0.10 ng hGH was detected in the medium, just above the detection limit of the ELISA. After stimulation with IBMX and Forskolin, 3.3±0.3 ng hGH is secreted in the medium, which is 79 times less than mGH. The remaining level of hGH in the cell lysate was 3.5±0.1 ng hGH (Figure 5E). The signal detected in all wild-type samples was under the detection limit, thereby excluding statistical comparison.

To assess whether hGH could be detected in the circulation, blood samples were taken at 3 different time points, namely 09:30 h, 10:00 h and 10:30 h, encompassing the peak of GH release in the mouse [17]. No hGH signal could be detected at any of the 3 time points in both wild-type and heterozygous samples (data not shown).

## Discussion

In this study we have delineated the dwarf phenotype in the *Nbea* ^+/*−*^ GH240B mouse model generated by Su *et al* [10]. We have established that *Nbea* ^+/*−*^ GH240B mice have an increased percentage of fat mass concomitant with a decrease in lean mass and bone mineral density. This phenotype is the result of alterations in the GH/IGF-1 axis caused by hGH expressed from the transgene in the pituitary and hypothalamus of *Nbea* ^+/*−*^ GH240B mice. GHRH levels in the hypothalamus are suppressed, resulting in reduced somatotroph stimulation, causing reduced cell numbers and mGH content.

When the transgenic construct of h*GH* was accidently inserted in intron 1 of *Nbea*, the absence of expression of hGH in the pituitary was verified by RT-PCR [10], [22]. Expression in other tissues was not examined. The discrepancy between the two *Nbea^+/−^* mouse models regarding dwarfism prompted us to reevaluate the expression of hGH, especially since 5 different transgenic mouse models were generated with the same transgenic h*GH* construct and analysis of pituitary mRNA revealed h*GH* expression in 3 of these mouse models [22]. Expression of hGH was unexpected since the two pituitary specific DNase I hypersensitive sites I and II (HS I and II), which are essential to drive high levels of somatotrope-specific hGH expression, were not incorporated in the transgenic construct [22]–[24]. hGH mRNA and protein were detected in the anterior pituitary and hypothalamus of *Nbea^+/−^* GH240B mice, using qRT-PCR and ELISA. Most likely, the absence of HS I and II resulted in a minimal expression of hGH reflecting the basal functions of the proximal regulatory elements of the h*GH* gene present in the transgenic construct [24].

The presence of hGH in the hypothalamus of *Nbea* ^+/*−*^ GH240B mice is probably at the basis of the observed GH deficiency and subsequent dwarfism. Other transgenic mouse models with targeted expression of hGH in the hypothalamus also present dwarfism and similar alterations at the level of the pituitary to *Nbea* ^+/*−*^ GH240B mice [25], [26]. The proposed molecular mechanism of their observed dwarf phenotype is a dominant negative feedback loop in which hGH is capable of binding the mGH receptor (mGHR) present in the hypothalamus [27], [28]. As a result, *Ghrh* mRNA in the hypothalamus is significantly reduced leading to a decrease in pituitary m*Gh* mRNA and protein levels [25]. Recently, a similar phenotype was observed in the AlfpCre mouse model in which the entire h*GH* gene was inserted into the AlfpCre transgene leading to the presence of hGH protein in the hypothalamus and pituitary [29], [30].

The 500-fold lower levels of hGH in the pituitary compared to mGH and the undetectable levels in the blood preclude a significant effect of hGH on IGF-1 production in liver, while the low expression level of hGH in hypothalamus is apparently sufficient to locally activate the mGHR and suppress GHRH expression. A possible explanation can be that, since mGH is not expressed in hypothalamus and can only activate the mGHR in this tissue through an endocrine mechanism, the hGH/mGH ratio in hypothalamus will probably be many times higher than in pituitary. However, we did not determine this ratio in the hypothalamus. No hGH was detected in the hippocampus of *Nbea* ^+/*−*^ GH240B mice, the brain region with the highest expression of Nbea, indicating that expression of hGH is not directed by the *Nbea* promoter but promoter elements in the h*GH* transgene itself, only activated in specific tissues. This is also consistent with the fact that the two genes are in opposite directions [10].

Similar to *Nbea* ^+/*−*^ GH240B mice, the total number of GH- and PRL-producing cells in the pituitary of mice with transgenic hGH expression in the hypothalamus is reduced whereas other cell types were unaffected leading to hypoplasia of the pituitary [26]. Pituitary hypoplasia is also present in other mouse models with affected GHRH signaling, more specifically the *Ghrhr* deficient *little* mice and the *Pc1/3* knockout mice in which the maturation of proGHRH is blocked [31], [32]. The selective reduction of GH and PRL-producing cells has also been observed in a transgenic rat model with hGH expression under the control of the *Ghrh* promoter [33]. The reduction of these two cell populations can be explained by developmental relationship and/or paracrine interplay [34].

The somatotrope cells present in the transgenic mice expressing hGH in the hypothalamus retain their functional capacity for GH secretion in *ex vivo* cell cultures both under basal condition and upon stimulation with GHRH [26]. In primary cultures of anterior pituitary cells of *Nbea* ^+/*−*^ GH240B mice, Forskolin and IBMX were applied as secretagogues mimicking the rise in cAMP caused by GHRHr activation [21]. A similar ratio of stimulated to basal GH release was measured for both genotypes indicating a normal responsiveness of the remaining GH-producing cells in *Nbea* ^+/*−*^ mice. The observed reduced presence of GH in the medium of cell cultures of *Nbea* ^+/*−*^ mice, both in basal and stimulated conditions, is most likely caused by the reduced presence of somatotrope cells in the anterior pituitary of *Nbea* ^+/*−*^ mice and the lower GH content per GH-producing cell. Since NBEA has been identified as a negative regulator of regulated secretion *in vitro* [35], the ratio of GH released upon stimulation versus basal release was anticipated to be higher in *ex vivo* cell cultures derived from *Nbea* ^+/*−*^ mice. A possible explanation for the similar ratios is that compensatory mechanisms in the somatotrope cell population of *Nbea* ^+/*−*^ GH240B mice mask any *ex vivo* effect of *Nbea* haploinsufficiency on regulated secretion.

For the assessment of the mRNA profile of members of the GH cascade, only the most relevant SST receptors were included, although 5 different SST receptors are present both in pituitary and hypothalamus [36]. In the arcuate nucleus of the hypothalamus, *Sstr4* and *5* were selected due to their predominant presence on neurons producing GHRH and Ghrelin [36]. In GH producing cells, *Sstr2* and *5* are most highly expressed [37]. Of note is that *Sstr2* and *5* are also expressed in thyrotrophs [38]. The observed decrease in *Ghrh* mRNA in the hypothalamus in combination with the increased mRNA level of *Sstr2* in the anterior pituitary might be at the basis of the reduced m*Gh* mRNA detected in the anterior pituitary of *Nbea* ^+/*−*^ mice. Most likely, in an attempt to compensate for the decrease in mGH, the mRNA level of *Ghr* is elevated in the anterior pituitary of *Nbea* ^+/*−*^ mice. The 85% reduction in m*Gh* in anterior pituitaries of *Nbea* ^+/*−*^ mice causes a 50% decrease in *Igf-1* mRNA in the liver and a 66% decrease in IGF-1 protein detected in plasma. Reduced plasma IGF-1 levels correlate with the dwarf phenotype and increased fat percentage [20].

The dwarf phenotype in the *Nbea* GH240B mouse model used in our experiments obfuscates the interpretation of body weight, although the body weight of 1-year-old *Nbea* ^+/*−*^ mice exceeds the body weight of *Nbea* ^+/+^ mice. Normal growth was reported for the *Nbea* ^+/*−*^ gene-trap mice and they also have an increased ratio of adipose tissue resulting in a higher body weight [15]. These findings suggest that the increase in adipose tissue is correlated with *Nbea* haploinsufficiency rather than solely with the method of generating the *Nbea* mouse model. However, it cannot be excluded that the gene-trap insertion has affected the expression of other genes involved in adipogenesis.

An important issue arising from this study is whether the mouse model is still useful for studying NBEA function and its role in ASD. The two models are highly similar with the exception of growth deficiency. In addition, behavioral studies with the GH240B mouse model have shown abnormalities in sociability, and spatial learning and memory and show an increase in conditioned fear and in repetitive self-grooming behavior, making this a highly promising model for the study of ASD [12]. However, we cannot exclude the fact that the disturbed GH/IGF-1 axis contributes to the behavioral abnormalities since low IGF-1 levels have been suggested to contribute to the development of ASD in a subgroup of patients [39], [40] and SHANK3-deficient ASD patients are being treated with IGF-1 in a clinical trial (Clinicaltrial.gov NCT01525901). On the other hand, no growth retardation nor IGF-1 deficiency has been reported in any of the NBEA haploinsufficient ASD patients and another study in children with ASD measured an increased level of IGF-1 instead of a decrease, making the influence of IGF-1 in ASD a controversial issue that should be further assessed [41]. Behavioral studies in the other Nbea knockout model and/or the mouse models expressing transgenic hGH should be performed to exclude the role of hGH in the behavioral phenotype. To the best of our knowledge, there are no documented patients with isolated congenital growth hormone deficiency that also have psychiatric disorders. Therefore, we assume that the ASD-like phenotype in our mouse model can be attributed to NBEA haploinsufficiency.

Another argument highlighting the usefulness of the GH240B model is that abnormalities in platelet morphology, which were first noticed in several ASD patients including one with NBEA haploinsufficiency, are also observed in platelets of this mouse model [11]. This study has also demonstrated an altered phosphorylation pattern of PKA substrates. Since Nbea is an AKAP, these results suggest that this mouse model may be most useful in the identification of PKA substrates whose phosphorylation is regulated by Nbea. The first such PKA substrate that was identified using this mouse model is the transcription factor CREB [12].

## Conclusion

This study has unveiled the molecular basis for the discrepancy between the two commonly used mouse models for NBEA deficiency. Despite the growth deficiency caused by recombinant hGH expression, the two *Nbea* mouse models are mostly comparable. Taken together, this correlation between the two mouse models, combined with correlations with the ASD patient and cell lines, gives credence to all findings regarding NBEA function. However, to exclude the confounding effects of hGH we recommend using the gene-trap mouse models in the future to study the physiological function of NBEA and its role in the pathogenesis of ASD.

## Supporting Information

Table S1 **Primers per gene used for quantitative reverse transcription PCR.** The sequences of the primers are presented in a 5′ to 3′ orientation. *Gh*: *growth hormone*; *Ghr*: *Gh receptor*; *Ghrh*: *Gh-releasing hormone*; *Ghrhr*: *Ghrh receptor*; *Sst*: *somatostatin*; *Sstr*: *Sst receptor*; *Ghrelinr*: *Ghrelin receptor*; h*GH*: human *GH*; *Igf-1*: *insulin-like growth factor 1*; *Gapdh*: *glyceraldehyde 3-phosphate dehydrogenase*.
(PDF)
