# Supplementary material for: The Dwarf Phenotype in GH240B Mice, Haploinsufficient for the Autism Candidate Gene Neurobeachin, Is Caused by Ectopic Expression of Recombinant Human Growth Hormone
Source: PLoS One. 2014 Oct 15;9(10):e109598. doi: 10.1371/journal.pone.0109598 (PMC4198124; doi:10.1371/journal.pone.0109598)
Supplement: Table S1 — Primers per gene used for quantitative reverse transcription PCR. The sequences of the primers are presented in a 5′ to 3′ orientation. Gh: growth hormone; Ghr: Gh receptor; Ghrh: Gh-releasing hormone; Ghrhr: Ghrh receptor; Sst: somatostatin; Sstr: Sst receptor; Ghrelinr: Ghrelin receptor; hGH: human GH; Igf-1: insulin-like growth factor 1; Gapdh: glyceraldehyde 3-phosphate dehydrogenase. (PDF) [file pone.0109598.s001.pdf]

Table S1: Primers per gene used for quantitative reverse transcription PCR.

	**Forward**	**Reverse**
Nbea	CTTTGTGCGGATCAACAGG	CATTGATGGAGTTGGCAAGA
Gh	CCTCAGCAGGATTTTCACCA	CTTGAGGATCTGCCCAACAC
Ghr	CCAGGATCTATTCAGCTGTACTATGC	TGGGTCCATTCATGAGCAATT
Ghrh	GCAGAACCTCAATCGGAGAG	TGGTGAGGATGAGGATCACA
Ghrhr	ACCCGTATCCTCTGCTTGCT	AGGTGTTGTTGGTCCCCTCT
Sst	CCCAGACTCCGTCAGTTTCT	GGGCATCATTCTCTGTCTGG
Sstr2	GAGGCCTTTCCCCTAGAGTT	CACCGTAACGCTTGTCCTT
Sstr4	TCTGCATCGTCCTGGCTTT	CTTGGCCAGTTCCTGTTTCC
Sstr5	TGGTCTTTGGGAAGGTGAAAG	TGTCCACAGTCGGAAATGGT
Ghrelin	CCAGAGGACAGAGGACAAGC	CATCGAAGGGAGCATTGAAC
Ghrelinr	GACCAGAACCACAAACAGACAG	GGCTCGAAAGACTTGGAAAA
hGH	CCAGGAGTTTGAAGAAGCCT	GGAGGTCATAGACGTTGCTGT
Igf-1	GCTCTTCAGTTCGTGTGTGGAC	CATCTCCAGTCTCCTCAGATC
Gapdh	ATGGCCTTCCGTGTTCCT	CAGGCGGCACGTCAGAT

The sequences of the primers are presented in a 5’ to 3’ orientation. *Gh*: *growth hormone*; *Ghr*: *Gh receptor*; *Ghrh*: *Gh-releasing hormone*; *Ghrhr*: *Ghrh receptor*; *Sst*: *somatostatin*; *Sstr*: *Sst receptor*; *Ghrelinr*: *Ghrelin receptor*; h*GH*: human *GH*; *Igf-1*: *insulin-like growth factor 1*; *Gapdh*: *glyceraldehyde 3-phosphate dehydrogenase*.
